# Optimal Degree of Hypothermia in Total Arch Replacement for Type A Aortic Dissection

**DOI:** 10.3389/fcvm.2021.668333

**Published:** 2021-04-28

**Authors:** Jinlin Wu, Juntao Qiu, Zhongrong Fang, Qipeng Luo, Yao Huang, Cuntao Yu, Guyan Wang

**Affiliations:** ^1^Department of Cardiac Surgery, Guangdong Cardiovascular Institute, Guangdong Provincial People's Hospital, Guangdong Academy of Medical Sciences, Guangzhou, China; ^2^Department of Vascular Surgery, Fuwai Hospital, National Center for Cardiovascular Diseases, Chinese Academy of Medical Sciences and Peking Union Medical College, Beijing, China; ^3^Department of Anesthesiology, Fuwai Hospital, National Center for Cardiovascular Diseases, Chinese Academy of Medical Sciences and Peking Union Medical College, Beijing, China; ^4^Department of Finance, HULT International Business School, San Francisco, CA, United States; ^5^Department of Anesthesiology, Beijing Tongren Hospital, Capital Medical University, Beijing, China

**Keywords:** aortic dissection, aortic aneurysm (thoracic), cardiopulmoanry bypass, hypothermia (induced), surgery

## Abstract

**Background:** We sought to investigate the best degree of hypothermic cardiac arrest (HCA) in type A aortic dissection (TAAD) with a cohort of 1,018 cases receiving total arch replacement from 2013 to 2018 in Fuwai Hospital.

**Method:** The cohort was divided by DHCA (≤24°C, *n* = 580) vs. MHCA (>24°C, *n* = 438), and interquartile range (Q1–Q4). Primary endpoints included mortality, stroke, paraplegia, and continuous renal replacement therapy (CRRT), which were summarized as composite major outcomes (CMO).

**Results:** The Odds Ratio (OR) of CMO for MHCA was 0.7 (95% CI: 0.5–1.0, *p* = 0.06) (unadjusted) and 0.6 (95% CI: 0.4–1.0, *p* = 0.055) (adjusted). DHCA group tended to have a significantly longer CPB time (175.6 ± 45.6 vs. 166.8 ± 49.8 min, *p* = 0.003), longer hospital stay (16.0 ± 13.6 vs. 13.5 ± 6.8 days, *p* < 0.001), and ICU stay [5.0 (3.9–6.6) vs. 3.8 (2.0–5.6) days]. A significantly greater blood loss was observed in DHCA group, with a greater requirement for RBC and platelet transfusion. Of note, MHCA showed a significant protective effect (60% risk reduction) for older patients (above 60 years) (OR 0.4; 95% CI: 0.2–0.8; *p* = 0.009). By quartering, Q1 had significantly higher mortality (10.9%) than Q4 (5.2%) (*p* = 0.035). For other comparisons, the gap was significantly widened in quartering between Q1 and Q4, i.e., the lower the temperature, the worse the outcomes, and vice versa. Propensity score matching and sensitivity analyses confirmed the above findings.

**Conclusions:** A paradigm change from DHCA to MHCA may be encouraged in TAAD arch operation, especially for the elderly.

## Introduction

Aortic surgery is still one of the most challenging operations in the cardiovascular field, especially when it involves the aortic arch, where we have to open the aorta and stop the circulation. Deep hypothermic cardiac arrest (DHCA) has been widely used in the aortic arch surgery. However, DHCA itself would also bring about problems such as coagulation dysfunction, systemic inflammatory response, and so on. The selective cerebral perfusion (SCP) made some researchers begin to question the necessity of DHCA. To this day, the debate on this issue continues. Some stated that deep hypothermia remained the gold standard for end-organ protection with circulatory arrest during aortic arch repair (1), while some held that moderate hypothermic cardiac arrest (MHCA) and SCP represented an effective strategy for the arch operation and might obviate the need for DHCA (2). There is still a scarcity of researches on the optimal degree of hypothermic cardiac arrest (HCA) for type A aortic dissection (TAAD).

In this study, we intended to make comprehensive head-to-head comparisons between DHCA and MHCA, to provide some new evidence on this issue with a large cohort of 1,018 cases.

## Methods

### Patients and Data Collection

The case-control study was reported in line with Strengthening the Reporting of Observational Studies in Epidemiology (STROBE) (3). The study was approved by the Chinese ethics committee, with informed consent not required due to its observational nature (Reference Number: ChiECRCT-20180041).

All consecutive patients admitted to the Aortic Surgery Department, Fuwai Hospital (Beijing, China) from January 2013 to December 2018 were enrolled prospectively into our database and form the basis of the current study. We retrieved the patients based on electronic operative notes. Anthropometric, radiologic, operative, and outcome data were manually accrued from individual electronic medical records and hospital charts. The enrollment was demonstrated in Supplementary Figure 1.

A total of 1,018 TAAD patients were entered into the final analysis. In view of the absence of a universally recognized standard terminology about definitions of mild, moderate, and deep hypothermia, we divided patients into two groups in our main analysis: DHCA (nasopharyngeal temperature ≤ 24°C, *n* = 580) vs. MHCA (nasopharyngeal temperature > 24°C, *n* = 438), based on previous work by Leshnower et al. (4). To avoid the potential bias caused by somewhat arbitrary dichotomy and show more details, we have also divided this cohort into four groups based on the nasopharyngeal temperature interquartile range (IQR): Q1 (13–19.6°C, *n* = 256), Q2 (19.7–23.1°C, *n* = 259), Q3 (23.2–25.5°C, *n* = 255), and Q4 (25.6–29.0°C, *n* = 248).

### Study Endpoints

Comorbid conditions and post-operative complications were defined using the Society of Thoracic Surgeons definitions, which is available online at http://www.sts.org/national-database. The primary endpoints were operative mortality, stroke, paraplegia, and continuous renal replacement therapy (CRRT). Operative mortality includes in-hospital mortality and 30-day mortality post-operatively. A parameter called composite major outcomes (CMO) was proposed, comprehensively focusing on survival, cerebral, spinal, and visceral protection.

The secondary endpoints were re-exploration for bleeding, tracheotomy, hospital stay, intensive care unit (ICU) stay, blood loss, and in-hospital blood product use. Long hospital stay and long ICU stay were determined with 75% IQR as the cutoff, which was 17 and 6 days, respectively.

### Operative Techniques

In this study, three types of TAR were performed: single TAR, TAR with frozen elephant trunk (TAR/FET), and TAR with aortic balloon occlusion (TAR/ABO). The detailed surgical techniques have been reported previously (5, 6).

### Statistical Analysis

Statistical analysis and data visualization were performed using the R 3.6.1 (R Foundation for Statistical Computing, Vienna, Austria). A two-tailed *p-*value of < 0.05 was considered statistically significant.

For continuous variables, the normally distributed data are expressed as mean ± standard deviation (SD), or median with IQR for the skewed data. Continuous data were evaluated for normality using the Kolmogorov–Smirnov-test. For dichotomy (MHCA vs. DHCA), student's *t*-test was used for normally distributed variables and Mann–Whitney *U-*test for non-normally distributed variables. When the nasopharyngeal temperature was divided into four groups according to IQR (by quartering), analysis of variance (ANOVA) or Kruskal–Wallis-test was performed. Categorical variables are presented as frequencies with percentages, and analyzed by the Chi-square test or Fisher's exact-test, as appropriate.

To adjust for unbalanced basic characteristics, a propensity score matching (PSM) analysis was performed with MHCA vs. DHCA using “matchit” package. All the variables in Table 1 (pre-operative data) were taken into consideration when doing the matching. PSM of 1:1 ratio by the “nearest neighbor” method was performed. A caliper equal to 0.20 standard deviations of logit distance measure (propensity score) was used. A total of 254 patients were discarded due to the impossibility to identify a suitable match, with 764 being well-matched. Also, a weighted PSM was performed to compare the four quartering groups, with matching weight being calculated by “trimatch” package. The matching weight method is an extension of inverse probability of treatment weighting (IPTW) that reweights both exposed and unexposed groups to emulate a propensity score matched population. Additionally, to get the adjusted Odds Ratio (OR) of CMO for MHCA, variables showing *p* < 0.05 in the univariable logistic regression analysis were entered in the multivariable model (backward stepwise logistic regression) using the original cohort. The best model was selected based on the Akaike information criterion (AIC).

**Table 1:** Pre-operative characteristics.

	**Total cohort**			***p***	**Propensity-matched data**			***p***
	**Overall**	**DHCA**	**MHCA**		**Overall**	**DHCA**	**MHCA**	
***n***	**1,018**	**580**	**438**		**764**	**382**	**382**	
Age (year, Mean ± SD)	49.1 ± 11.4	46.7 ± 10.7	52.3 ± 11.7	<0.001	50.1 ± 10.7	49.7 ± 10.2	50.5 ± 11.3	0.310
Age ≥ 60 year	202 (19.8)	69 (11.9)	133 (30.4)	<0.001	152 (19.9)	65 (17.0)	87 (22.8)	0.057
Male (%)	760 (74.7)	447 (77.1)	313 (71.5)	0.050	572 (74.9)	287 (75.1)	285 (74.6)	0.934
BMI (kg/m^2^, Mean ± SD)	26.0 ± 4.5	26.0 ± 4.7	25.9 ± 4.4	0.686	26.0 ± 4.2	26.0 ± 4.0	26.0 ± 4.4	0.917
Hypertension (%)	814 (80.0)	445 (76.7)	369 (84.2)	0.004	631 (82.6)	314 (82.2)	317 (83.0)	0.849
Diabetes mellitus (%)	30 (2.9)	14 (2.4)	16 (3.7)	0.332	22 (2.9)	11 (2.9)	11 (2.9)	1.000
Coronary artery disease (%)	28 (2.8)	14 (2.4)	14 (3.2)	0.574	22 (2.9)	11 (2.9)	11 (2.9)	1.000
COPD (%)	6 (0.6)	1 (0.2)	5 (1.1)	0.113	2 (0.3)	1 (0.3)	1 (0.3)	1.000
Marfan syndrome (%)	91 (8.9)	73 (12.6)	18 (4.1)	<0.001	39 (5.1)	21 (5.5)	18 (4.7)	0.742
Smoking (%)	421 (41.4)	232 (40.0)	189 (43.2)	0.344	321 (42.0)	157 (41.1)	164 (42.9)	0.660
Family Hx of AD (%)	20 (2.0)	16 (2.8)	4 (0.9)	0.061	6 (0.8)	2 (0.5)	4 (1.0)	0.682
Hx of cardiac surgery (%)	53 (5.2)	35 (6.0)	18 (4.1)	0.220	36 (4.7)	19 (5.0)	17 (4.5)	0.864
Hx of aortic surgery (%)	53 (5.2)	30 (5.2)	23 (5.3)	1.000	40 (5.2)	19 (5.0)	21 (5.5)	0.871
Onset within 14 days (%)	855 (84.0)	486 (83.8)	369 (84.2)	0.913	636 (83.2)	314 (82.2)	322 (84.3)	0.498
HB (g/L, mean ± SD)	135.7 ± 17.4	135.4 ± 17.7	136.0 ± 17.0	0.613	135.2 ± 17.1	135.1 ± 17.4	135.3 ± 16.9	0.825
WBC (10^9^/L, mean ± SD)	11.4 ± 4.9	11.4 ± 5.2	11.5 ± 4.4	0.600	11.4 ± 5.1	11.3 ± 5.8	11.5 ± 4.4	0.737
PLT (10^9^/L, mean ± SD)	195.5 ± 78.4	194.2 ± 78.9	197.3 ± 77.8	0.526	195.6 ± 79.6	196.0 ± 83.7	195.2 ± 75.3	0.891
Penn classification^*^ (%)				0.147				0.869
Penn class Aa	761 (74.8)	418 (72.1)	343 (78.3)		585 (76.6)	293 (76.7)	292 (76.4)	
Penn class Ab	229 (22.5)	144 (24.8)	85 (19.4)		162 (21.2)	82 (21.5)	80 (20.9)	
Penn class Ac	21 (2.1)	13 (2.2)	8 (1.8)		13 (1.7)	5 (1.3)	8 (2.1)	
Penn class Ab&c	7 (0.7)	5 (0.9)	2 (0.5)		4 (0.5)	2 (0.5)	2 (0.5)	

* *Penn Classification: Penn Class Aa (No ischemia), Penn Class Ab (Localized ischemia), Penn Class Ac (Generalized ischemia/circulatory collapse), Penn Class Ab&c (Combined ischemia). DHCA, deep hypothermic cardiac arrest; MHCA, moderate hypothermic cardiac arrest; SD, standard deviation; BMI, body mass index; COPD, chronic obstructive pulmonary disease; AD, aortic disease; Hx, history; HB, hemoglobin; WBC, white blood cell; PLT, platelet*.

## Results

### Pre-operative Characteristics of Patients

The clinical characteristics of the 1,018 patients were shown in Table 1, stratified by DHCA (*n* = 580, 56.9%) and MHCA (*n* = 438, 43.1%). The mean age at presentation was 46.7 ± 10.7 and 52.3 ± 11.7 years for DHCA and MHCA group, respectively (*p* < 0.001). The percentage of male was 447 (77.1%) and 313 (71.5%) for DHCA and MHCA group, respectively (*p* = 0.050). We presented malperfusion with Penn Classification (7), and no significant difference was found. Altogether, 382 pairs of patients were matched using PSM. The love plot (Supplementary Figure 2) demonstrated well-balanced absolute standardized differences between the two groups regarding baseline characteristics, consistent with the right half of Table 1. By quartering, Supplementary Table 1 shows similar information to Table 1.

### Operative Data

**Figure 1:**
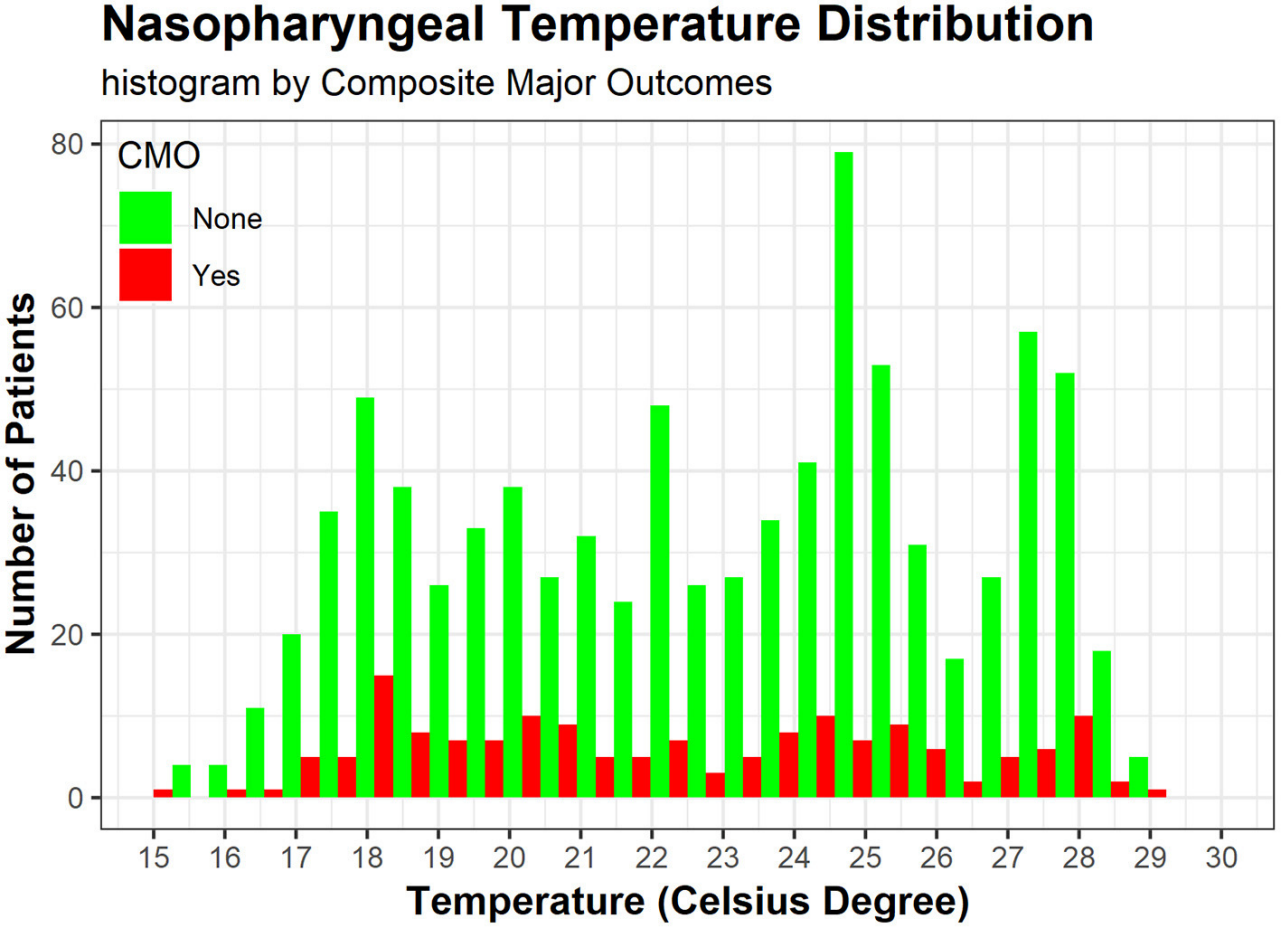
The histogram shows nasopharyngeal temperature distribution by CMO (red) and not (green), both of which display a bimodal distribution around 18 and 25°C. CMO, composite major outcomes, including operative mortality, stroke, paraplegia, and CRRT (continuous renal replacement therapy).

The operative details for DHCA vs. MHCA were summarized in Table 2. Compared to MHCA group, DHCA group tended to have a significantly longer CPB time (175.6 ± 45.6 vs. 166.8 ± 49.8 min, *p* = 0.003) and DHCA time (21.0 ± 7.0 vs. 14.7 ± 5.7 min, *p* < 0.001). The nasopharyngeal temperature for DHCA and MHCA group was 20.2 ± 2.2 and 26.2 ± 1.4°C (*p* < 0.001), respectively. As is shown in Figure 1, The nasopharyngeal temperature ranged from 13 to 29°C, ensuring enough events and samples within each temperature interval, forming a good basis for our research.

**Table 2:** Operative data.

	**Total cohort**			***p***	**Propensity-matched data**			***p***
	**Overall**	**DHCA**	**MHCA**		**Overall**	**DHCA**	**MHCA**	
***n***	**1,018**	**580**	**438**		**764**	**382**	**382**	
Root operation (%)				0.003				0.251
Bentall	253 (24.9)	167 (28.8)	86 (19.6)		160 (20.9)	88 (23.0)	72 (18.8)	
Root-sparing	739 (72.6)	403 (69.5)	336 (76.7)		579 (75.8)	285 (74.6)	294 (77.0)	
David	9 (0.9)	4 (0.7)	5 (1.1)		9 (1.2)	4 (1.0)	5 (1.3)	
Wheat's	17 (1.7)	6 (1.0)	11 (2.5)		16 (2.1)	5 (1.3)	11 (2.9)	
Arch operation (%)				<0.001				<0.001
TAR	22 (2.2)	14 (2.4)	8 (1.8)		17 (2.2)	12 (3.1)	5 (1.3)	
TAR/FET	916 (90.0)	556 (95.9)	360 (82.2)		678 (88.7)	363 (95.0)	315 (82.5)	
TAR/ABO	80 (7.9)	10 (1.7)	70 (16.0)		69 (9.0)	7 (1.8)	62 (16.2)	
CABG (%)	106 (10.4)	55 (9.5)	51 (11.6)	0.311	77 (10.1)	33 (8.6)	44 (11.5)	0.229
Ascending-iliac bypass (%)	59 (5.8)	29 (5.0)	30 (6.8)	0.265	42 (5.5)	18 (4.7)	24 (6.3)	0.427
Operation time (hour, Mean ± SD)	6.2 ± 1.7	6.2 ± 1.5	6.3 ± 1.9	0.354	6.2 ± 1.7	6.2 ± 1.4	6.3 ± 2.0	0.204
CPB time (min, Mean ± SD)	171.8 ± 47.6	175.6 ± 45.6	166.8 ± 49.8	0.003	170.3 ± 47.2	174.5 ± 44.2	166.0 ± 49.7	0.012
HCA time (min, Mean ± SD)	18.3 ± 7.2	21.0 ± 7.0	14.7 ± 5.7	<0.001	17.8 ± 7.1	20.8 ± 7.1	14.7 ± 5.7	<0.001
HCA > 22 min (%)	227 (22.3)	200 (34.5)	27 (6.2)	<0.001	150 (19.6)	128 (33.5)	22 (5.8)	<0.001
Nadir temperature (°C, Mean ± SD)	22.8 ± 3.5	20.2 ± 2.2	26.2 ± 1.4	<0.001	23.1 ± 3.4	20.2 ± 2.2	26.1 ± 1.4	<0.001

*DHCA, deep hypothermic cardiac arrest; MHCA, moderate hypothermic cardiac arrest; TAR, total arch replacement; FET, frozen elephant trunk; ABO, aortic balloon occlusion; CABG, coronary artery bypass graft; SD, standard deviation; CPB, cardiopulmonary bypass; HCA, hypothermic cardiac arrest*.

Of note, some subtle information was revealed by quartering (Supplementary Table 1): Q1 (13–19.6°C) interval had an obvious longer operation time (6.6 ± 1.4 vs. 6.2 ± 1.8 h, *p* < 0.001) and CPB time (187.6 ± 47.5 vs. 170.4 ± 51.4 min, *p* < 0.001) compared to Q4 (25.6–29.0°C) interval. The weighted results further strengthened the above findings (Supplementary Table 2).

### Outcome Data

The post-operative details are summarized in Table 3. Overall, the CMO, operative mortality, stroke, paraplegia, and CRRT rates were 160 (15.7%), 72 (7.1%), 30 (2.9%), 33 (3.2%), and 85 (8.3%), respectively. Although an advantageous trend was observed for MHCA over DHCA concerning the aforementioned primary endpoints, no statistical differences were reached. For secondary endpoints, DHCA group had a significantly longer hospital stay (16.0 ± 13.6 vs. 13.5 ± 6.8 days, *p* < 0.001) and ICU stay [5.0 (IQR: 3.9–6.6) vs. 3.8 (IQR: 2.0–5.6) days] compared with MHCA group. A significantly greater blood loss was observed in DHCA group [780 (IQR: 600–1,200) vs. 720 (IQR: 600–900) ml, *p* = 0.004] compared to MHCA group, with a greater requirement for RBC transfusion [4 (IQR: 0–8) vs. 2 (IQR: 0–6) unit, *p* = 0.009] and platelet transfusion [4 (IQR: 3–4) vs. 1 (IQR: 1–2) unit, *p* < 0.001].

**Table 3:** Outcome data.

	**Total cohort**			***p***	**Propensity-matched data**			***p***
	**Overall**	**DHCA**	**MHCA**		**Overall**	**DHCA**	**MHCA**	
***n***	**1,018**	**580**	**438**		**764**	**382**	**382**	
CMO (%)	160 (15.7)	102 (17.6)	58 (13.2)	0.072	113 (14.8)	64 (16.8)	49 (12.8)	0.154
Operative mortality (%)	72 (7.1)	49 (8.4)	23 (5.3)	0.065	51 (6.7)	31 (8.1)	20 (5.2)	0.147
Stroke (%)	30 (2.9)	18 (3.1)	12 (2.7)	0.879	22 (2.9)	11 (2.9)	11 (2.9)	1.000
Paraplegia (%)	33 (3.2)	23 (4.0)	10 (2.3)	0.186	24 (3.1)	16 (4.2)	8 (2.1)	0.147
CRRT (%)	85 (8.3)	54 (9.3)	31 (7.1)	0.246	62 (8.1)	36 (9.4)	26 (6.8)	0.233
Reexploration for bleeding (%)	38 (3.7)	25 (4.3)	13 (3.0)	0.341	25 (3.3)	15 (3.9)	10 (2.6)	0.416
Tracheotomy (%)	37 (3.6)	19 (3.3)	18 (4.1)	0.593	31 (4.1)	16 (4.2)	15 (3.9)	1.000
Hospital stay (day, mean ± SD)	14.9 ± 11.2	16.0 ± 13.6	13.5 ± 6.8	<0.001	14.7 ± 11.2	15.9 ± 14.3	13.5 ± 6.7	0.003
Hospital stay >17 days (%)	245 (24.1)	155 (26.7)	90 (20.5)	0.027	180 (23.6)	103 (27.0)	77 (20.2)	0.033
ICU stay [day, median (IQR)]	4.6 (3.5–6)	5.0 (3.9–6.6)	3.8 (2.0–5.6)	0.002	4.6 (3–6)	4.9 (3.9–6.5)	4 (2–5.6)	0.027
ICU stay > 6 days (%)	252 (24.8)	166 (28.6)	86 (19.6)	0.001	175 (22.9)	99 (25.9)	76 (19.9)	0.058
Blood loss [ml, median (IQR)]	750 (600–960)	780 (600–1,200)	720 (600–900)	0.004	720 (600–900)	765 (600–1,200)	720 (600–900)	0.029
**In-hospital blood product use**								
RBC [unit, median (IQR)]	4 (0–6)	4 (0–8)	2 (0–6)	0.009	4 (0–6)	4 (0–8)	2 (0–6)	0.013
Plasma [ml, median (IQR)]	400 (0–800)	400 (0–800)	400 (0–800)	0.935	400 (0–800)	400 (0–800)	400 (0–800)	0.691
PLT [unit, median (IQR)]	3 (1–4)	4 (3–4)	1 (1–2)	<0.001	2 (1–4)	4 (3–4)	1 (1–2)	<0.001

*DHCA, deep hypothermic cardiac arrest; MHCA, moderate hypothermic cardiac arrest; CMO, composite major outcomes; CRRT, continuous renal replacement therapy; SD, standard deviation; ICU, intensive care unit; IQR, interquartile range; RBC, red blood cell; PLT, platelet*.

PSM confirmed the above findings. By quartering (Supplementary Table 1), Q1 (13–19.6°C) interval had significantly higher operative mortality [28 (10.9%) vs. 13 (5.2%), *p* = 0.035] than Q4 (25.6–29.0°C) interval. Q2 (19.7–23.1°C) had the highest paraplegia rate [16 (6.2%)] compared to other intervals (*p* = 0.02). For other outcomes of significance in dichotomy (DHCA vs. MHCA), the gap was significantly widened in quartering between Q1 and Q4. The weighted results further strengthened the above findings (Supplementary Table 2).

### Other Analyses

To identify independent predictors of CMO, we performed univariable logistic analyses followed by stepwise backward multivariable logistic analysis. The model showed that advanced age (per 10 years) (OR 1.3; 95% CI: 1.1–1.5; *p* = 0.001), malperfusion (Penn Classification Ab, Ac, or Ab&c) (OR 1.7; 95% CI: 1.2–2.5; *p* = 0.005), ascending-iliac bypass procedure (OR 2.3; 95% CI: 1.1–4.4; *p* = 0.013) and long operation time (per hour) (OR 1.4; 95% CI: 1.3–1.5; *p* < 0.001) were independent risk predictor for CMO, while male (OR 0.6; 95% CI: 0.4–0.9; *p* = 0.022) and higher pre-operative platelet level (^*^10^9^/L) (OR 0.9; 95% CI: 0.9–0.9; *p* = 0.009) were independent protective predictor for CMO. In this model, MHCA showed a trend of protective effect on CMO (adjusted OR 0.6; 95% CI: 0.4–1.0). However, it narrowly missed the significant point (*p* = 0.055).

**Figure 2:**
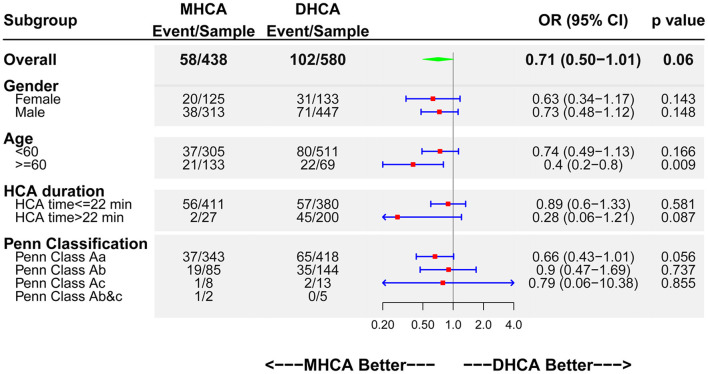
Forest plot of MHCA vs. DHCA regarding CMO by subgroups. DHCA, deep hypothermic cardiac arrest; MHCA, moderate hypothermic cardiac arrest; CMO, composite major outcomes; HCA, hypothermic cardiac arrest; OR, odds ratio; CI, confidence interval.

A subgroup analysis (CMO as endpoint) was performed (Figure 2). It showed that MHCA had an advantage over DHCA irrespective of the subgroup stratifications. Interestingly, MHCA showed a significant protective effect (up to 60% risk reduction) for older patients (above 60 years) (OR 0.4; 95% CI: 0.2–0.8; *p* = 0.009).

**Figure 3:**
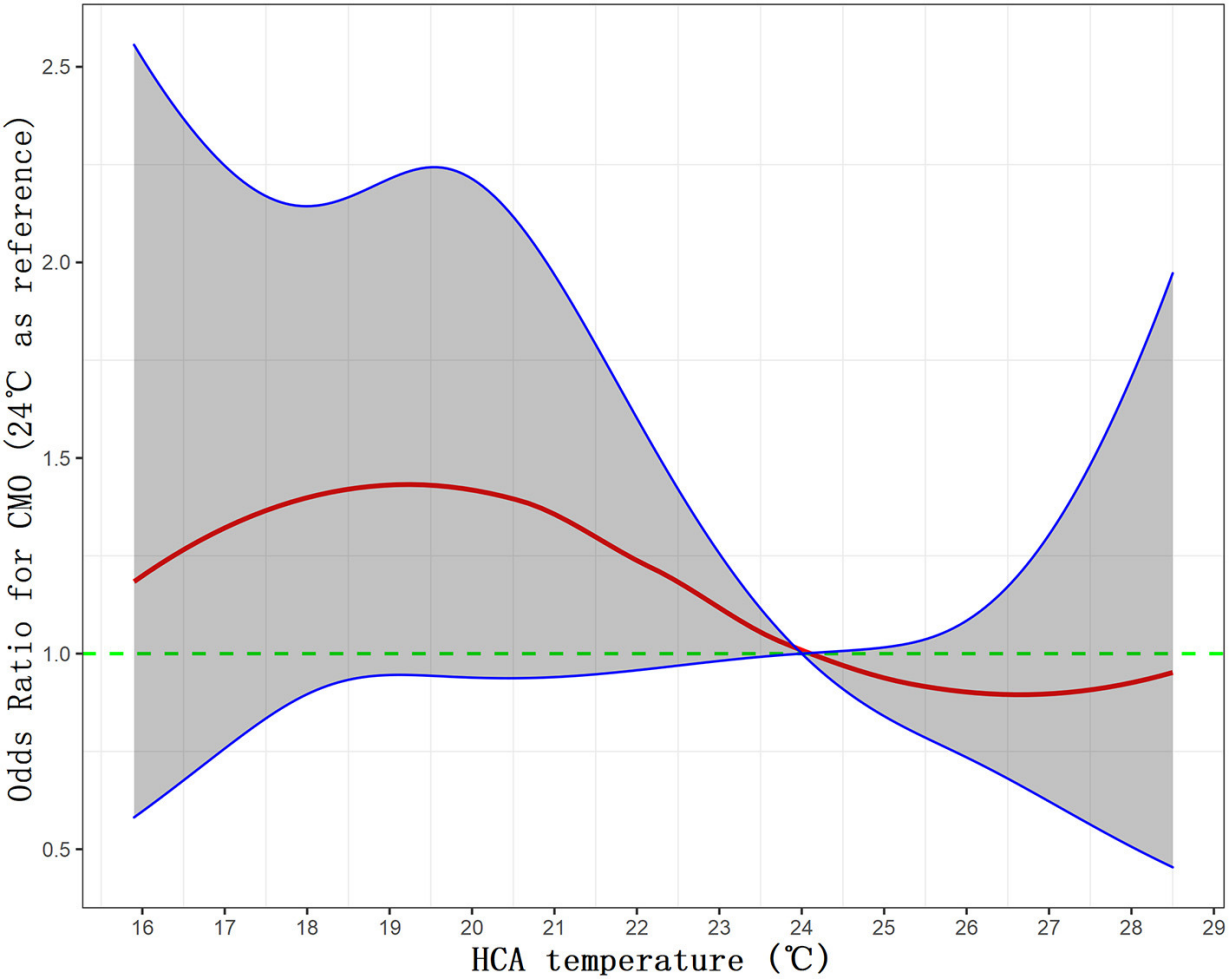
Visualization of the relationship between HCA and CMO by restricted cubic splines to the logistic model. CMO, composite major outcomes; HCA, hypothermic cardiac arrest.

To further delineate the relationship between HCA temperature and COM, we have used restricted cubic splines to logistic model and visualized the relation of HCA temperature with CMO, in which the temperature was taken as a continuous variable as it is rather than a categorical variable (Figure 3). Overall, the plot showed a reduction of the risk within the upper range (the risk of CMO below 24°C was higher than that of above), which was consistent with our previous findings by dichotomy. Interestingly, a non-linear relationship was revealed between the HCA temperature and the risk of CMO. The risk peaked at 19°C, not the lowest temperature (16°C in this plot). The risk touched bottom at 26.5°C and seemed to ascend again when the temperature exceeded 28°C.

## Discussion

To our best literature review, this study has been the largest single-center cohort on the issue of optimal degree of HCA in TAAD, representing a relatively homogenous study population. Our main finding was that compared with DHCA, MHCA was inclined to be a protective factor for primary endpoints (CMO, operative mortality, stroke, paraplegia, and CRRT), although it narrowly missed the significant point. For secondary endpoints, MHCA outperformed DHCA significantly regarding hospital stay, ICU stay, blood loss, and in-hospital blood product use. Of note, by quartering, the Q1 (13.0–19.6°C) interval had significantly higher operative mortality [28 (10.9%) vs. 13 (5.2%), *p* = 0.035] than the Q4 (25.6–29.0°C) interval. For outcomes significant in the dichotomy analyses, the gap was significantly widened in quartering between Q1 and Q4, i.e., the lower the temperature, the worse the outcomes and vice versa (within a range of 13–29°C). Also, DHCA group had longer operation time and CPB time compared with MHCA, which have been well-established risk factors for aortic adverse events. These results favor MHCA over DHCA in the setting of TAAD. Restricted cubic spline analysis revealed a similar trend, with 19°C being the peak risk point, and 26.5°C the bottom risk point.

Our results were supported by Leshnower et al. (4). They reported a series of 288 TAAD patients, in which 88 patients underwent DHCA (21.6 ± 3.1°C) and 206 patients underwent MHCA (27.4 ± 1.6°C). Mortality was 14.6% for DHCA patients, and 9.2% for MHCA patients (*p* = 0.17). Although no significant difference in stroke and dialysis-dependent renal failure was found, they noticed an advantageous trend for MHCA.

The major concern about MHCA is poor cerebral protection. In theory, DHCA should have a better protective effect on the brain than MHCA, because DHCA can reduce the metabolic rate of the brain to a smaller level, thus prolonging the ischemic tolerance period. Confirmed by animal model (8), at 21°C, hippocampal neurons have been shown to survive for 15 h in anoxic conditions, significantly more than the 5 h afforded at 28°C. In the absence of glucose and oxygen, cooling to 21°C confers a 300% increase in neuronal survival time compared to 28°C. Our results showed that MHCA had the same effect as DHCA on brain protection, with a stroke rate of 2.7 vs. 3.1% (*p* = 0.879). SCP is supposed to play an essential role here. In a prospective randomized trial (9) comparing DHCA alone and MHCA couple with SCP, from before to after arrest, jugular bulb pO_2_ changed by −21.67 mm Hg (26.4) in the DHCA group vs. +2.27 mm Hg (18.8) in the MHCA/SCP group (*p* = 0.007). Oxygen extraction changed by +1.7 mL/dL (1.3) in the DHCA group vs. −1 mL/dL (2.4) in the MHCA/SCP group (*p* < 0.001). Therefore, it is not surprising that MHCA/SCP is comparable with DHCA/SCP on the protection of the brain. Then, another question ensues-how about visceral protection, which doesn't have SCP during HCA? The indicator of visceral protection in this study is CRRT. Although not statistically significant, the CRRT rate in MHCA group is even lower than that of DHCA group (7.1 vs. 9.3%, *p* = 0.246). We speculated several reasons as follows: First, DHCA emphasizes more on brain protection. Internal organs like kidneys have better tolerance to ischemia than the brain, so the protective effect of MHCA may be good enough. Second, the HCA duration in our center is generally short (18.3 ± 7.2 min), which is also believed to be the experience at other high-volume centers, rendering the DHCA unnecessary. Third, DHCA does not confer much physiological benefit when it is weighed against the prolonged operation time, CPB time, and a greater requirement for blood transfusion, all of which are well-established risk factors for post-operative renal failure.

It's interesting to note that in our subgroup analysis, MHCA showed a significant protective effect (up to 60% risk reduction) for older patients (above 60 years) (OR 0.4; 95% CI: 0.2–0.8; *p* = 0.009). A possible explanation is that older people have poorer body reserves than younger people and hence are more susceptible to the potential blow by DHCA such as systemic inflammatory response and coagulopathy. Given the global trend of aging, especially in developed countries, we would encounter more and more older TAAD patients. In this setting, a paradigm shift from DHCA to MHCA is of greater clinical value and should be encouraged.

### Study Limitations

The study is observational and retrospective in nature. However, we had a good sample size and performed a variety of statistical methods, including multivariable regression, PSM, and subgroup analysis to reduce the impact of potential bias. Although randomized controlled trials might be a better clinical design, its ethical justification should be thoroughly discussed considering those evidence against DHCA.

### Conclusion

The treatment for TAAD is still very challenging, with high operative mortality and complications. An evolution toward MHCA for aortic arch surgery is currently ongoing in the global practice. The current robust evidence further strengthens this evolution. Our study shows that MHCA combined with SCP is a safe and effective technique for TAAD, demonstrating a non-inferior, even superior trend over DHCA regarding mortality, cerebral, spinal and visceral protection. Besides, MHCA had shorter operation time, CPB time, hospital stay and ICU stay, and less blood transfusion requirement compared to DHCA. Therefore, a paradigm change from DHCA to MHCA may be encouraged in TAAD arch operation, which may further improve the outcomes, especially for the elderly.

## Data Availability Statement

According to the policy of our institute, the data were not allowed to be made public. Requests to access these datasets should be directed to the corresponding authors.

## Ethics Statement

The studies involving human participants were reviewed and approved by Chinese Ethics Committee. Written informed consent for participation was not required for this study in accordance with the national legislation and the institutional requirements.

## Author Contributions

JW, CY, and GW: conception and design. JW and CY: overall responsibility. CY: obtained funding. JW and YH: statistical analysis. CY and GW: final approval of the article. YH and ZF: critical revision of the article. JW: writing the article. JW, JQ, QL, and ZF: data collection. JW and JQ: analysis and interpretation. All authors contributed to the article and approved the submitted version.

## Conflict of Interest

The authors declare that the research was conducted in the absence of any commercial or financial relationships that could be construed as a potential conflict of interest.
