# Supplementary material for: Optimal Degree of Hypothermia in Total Arch Replacement for Type A Aortic Dissection
Source: Front Cardiovasc Med. 2021 Apr 28;8:668333. doi: 10.3389/fcvm.2021.668333 (PMC8115724; doi:10.3389/fcvm.2021.668333)
Supplement: Supplementary file 1 [file Data_Sheet_1.docx]

**Supplemental Table 1. Preoperative, Operative and Outcome data by IQR**

|  | **Overall** | **HCA_Q1 [13,19.6]** | **HCA_Q2 (19.6,23.1]** | **HCA_Q3 (23.1,25.5]** | **HCA_Q4 (25.5,29]** | **p** |
| --- | --- | --- | --- | --- | --- | --- |
| **n** | **1018** | **256** | **259** | **255** | **248** |  |
| **Preoperative characteristics** |  |  |  |  |  |  |
| **Age (year, Mean±SD)** | 49.1 ±11.4 | 47.1 ±10.1 | 46.9 ±11.2 | 47.8 ±10.8 | 54.9 ±11.7 | <0.001 |
| **Age>=60 year** | 202 (19.8) | 26 (10.2) | 39 (15.1) | 37 (14.5) | 100 (40.3) | <0.001 |
| **Male (%)** | 760 (74.7) | 191 (74.6) | 201 (77.6) | 198 (77.6) | 170 (68.5) | 0.063 |
| **BMI (kg/m^2, Mean±SD)** | 26.0 ±4.5 | 25.7 ±4.3 | 25.9 ±4.7 | 26.2 ±4.4 | 26.0 ±4.7 | 0.662 |
| **Hypertension (%)** | 814 (80.0) | 191 (74.6) | 203 (78.4) | 209 (82.0) | 211 (85.1) | 0.022 |
| **Diabetes Mellitus (%)** | 30 (2.9) | 2 (0.8) | 9 (3.5) | 8 (3.1) | 11 (4.4) | 0.094 |
| **Coronary Artery Disease (%)** | 28 (2.8) | 7 (2.7) | 6 (2.3) | 7 (2.7) | 8 (3.2) | 0.942 |
| **COPD (%)** | 6 (0.6) | 1 (0.4) | 0 (0.0) | 2 (0.8) | 3 (1.2) | 0.32 |
| **Marfan Syndrome (%)** | 91 (8.9) | 28 (10.9) | 41 (15.8) | 15 (5.9) | 7 (2.8) | <0.001 |
| **Smoking (%)** | 421 (41.4) | 104 (40.6) | 102 (39.4) | 110 (43.1) | 105 (42.3) | 0.824 |
| **Family Hx of AD (%)** | 20 (2.0) | 7 (2.7) | 8 (3.1) | 2 (0.8) | 3 (1.2) | 0.167 |
| **Hx of Cardiac Surgery (%)** | 53 (5.2) | 12 (4.7) | 19 (7.3) | 6 (2.4) | 16 (6.5) | 0.057 |
| **Hx of Aortic Surgery (%)** | 53 (5.2) | 11 (4.3) | 18 (6.9) | 8 (3.1) | 16 (6.5) | 0.171 |
| **Onset within 14 days (%)** | 855 (84.0) | 214 (83.6) | 215 (83.0) | 221 (86.7) | 205 (82.7) | 0.594 |
| **HB (g/L, Mean±SD)** | 135.7 ±17.4 | 134.5 ±17.9 | 135.5 ±17.9 | 137.2 ±16.2 | 135.4 ±17.5 | 0.347 |
| **WBC (10^9/L, Mean±SD)** | 11.4 ±4.9 | 11.2 ±3.8 | 11.5 ±6.6 | 11.5 ±3.9 | 11.5 ±4.7 | 0.853 |
| **PLT (10^9/L, Mean±SD)** | 195.5 ±78.4 | 188.3 ±68.4 | 195.5 ±75.8 | 199.5 ±90.0 | 198.9 ±78.1 | 0.353 |
| **Penn Classification* (%)** |  |  |  |  |  | 0.192 |
| **Penn Class Aa** | 761 (74.8) | 183 (71.5) | 186 (71.8) | 187 (73.3) | 205 (82.7) |  |
| **Penn Class Ab** | 229 (22.5) | 65 (25.4) | 65 (25.1) | 61 (23.9) | 38 (15.3) |  |
| **Penn Class Ac** | 21 (2.1) | 5 (2.0) | 6 (2.3) | 6 (2.4) | 4 (1.6) |  |
| **Penn Class Ab&c** | 7 (0.7) | 3 (1.2) | 2 (0.8) | 1 (0.4) | 1 (0.4) |  |
| **Operative data** |  |  |  |  |  |  |
| **Root Operation (%)** |  |  |  |  |  | 0.02 |
| **Bentall** | 253 (24.9) | 71 (27.7) | 82 (31.7) | 57 (22.4) | 43 (17.3) |  |
| **Root-sparing** | 739 (72.6) | 181 (70.7) | 173 (66.8) | 190 (74.5) | 195 (78.6) |  |
| **David** | 9 (0.9) | 1 (0.4) | 2 (0.8) | 3 (1.2) | 3 (1.2) |  |
| **Wheat's** | 17 (1.7) | 3 (1.2) | 2 (0.8) | 5 (2.0) | 7 (2.8) |  |
| **Arch Operation (%)** |  |  |  |  |  | <0.001 |
| **TAR** | 22 (2.2) | 7 (2.7) | 6 (2.3) | 6 (2.4) | 3 (1.2) |  |
| **TAR/FET** | 916 (90.0) | 248 (96.9) | 248 (95.8) | 225 (88.2) | 195 (78.6) |  |
| **TAR/ABO** | 80 (7.9) | 1 (0.4) | 5 (1.9) | 24 (9.4) | 50 (20.2) |  |
| **CABG (%)** | 106 (10.4) | 34 (13.3) | 16 (6.2) | 23 (9.0) | 33 (13.3) | 0.019 |
| **Ascending-Iliac Bypass (%)** | 59 (5.8) | 11 (4.3) | 16 (6.2) | 15 (5.9) | 17 (6.9) | 0.651 |
| **Operation Time (hour, Mean±SD)** | 6.2 ±1.7 | 6.6 ±1.4 | 5.8 ±1.5 | 6.3 ±1.9 | 6.2 ±1.8 | <0.001 |
| **CPB Time (min, Mean±SD)** | 171.8 ±47.6 | 187.6 ±47.5 | 167.0 ±38.8 | 162.2 ±48.4 | 170.4 ±51.4 | <0.001 |
| **HCA Time (min, Mean±SD)** | 18.3 ±7.2 | 23.0 ±7.7 | 20.0 ±6.2 | 15.7 ±5.4 | 14.4 ±6.0 | <0.001 |
| **HCA Time>22 min (%)** | 227 (22.3) | 118 (46.1) | 72 (27.8) | 22 (8.6) | 15 (6.0) | <0.001 |
| **Nadir Temperature (℃, Mean±SD)** | 22.8 ±3.5 | 18.1 ±1.0 | 21.4 ±1.0 | 24.5 ±0.6 | 27.2 ±0.9 | <0.001 |
| **Clinical Outcomes** |  |  |  |  |  |  |
| **CMO (%)** | 160 (15.7) | 49 (19.1) | 42 (16.2) | 36 (14.1) | 33 (13.3) | 0.273 |
| **Operative Mortality (%)** | 72 (7.1) | 28 (10.9) | 18 (6.9) | 13 (5.1) | 13 (5.2) | 0.035 |
| **Stroke (%)** | 30 (2.9) | 7 (2.7) | 6 (2.3) | 8 (3.1) | 9 (3.6) | 0.841 |
| **Paraplegia (%)** | 33 (3.2) | 5 (2.0) | 16 (6.2) | 7 (2.7) | 5 (2.0) | 0.02 |
| **CRRT (%)** | 85 (8.3) | 30 (11.7) | 20 (7.7) | 16 (6.3) | 19 (7.7) | 0.138 |
| **Reexploration for bleeding (%)** | 38 (3.7) | 12 (4.7) | 10 (3.9) | 10 (3.9) | 6 (2.4) | 0.598 |
| **Tracheotomy (%)** | 37 (3.6) | 12 (4.7) | 6 (2.3) | 9 (3.5) | 10 (4.0) | 0.529 |
| **Hospital Stay (day, Mean±SD)** | 14.9±11.2 | 17.9±17.7 | 14.6±9.3 | 13.5±6.6 | 13.6±6.8 | <0.001 |
| **>17 days (%)** | 245 (24.1) | 87 (34.0) | 54 (20.8) | 50 (19.6) | 54 (21.8) | <0.001 |
| **ICU Stay (day, median (IQR))** | 4.6 (3.5-6) | 5.0 (4-6.7) | 4.8 (3.9-6.5) | 4 (2-5.6) | 4 (2-5.8) | <0.001 |
| **>6 days (%)** | 252 (24.8) | 83 (32.4) | 72 (27.8) | 46 (18.0) | 51 (20.6) | <0.001 |
| **Blood Loss (ml, median (IQR))** | 750 (600-960) | 900 (600-1200) | 630 (600-1080) | 780 (600-900) | 690 (600-900) | <0.001 |
| **In-Hospital Blood Product Use** |  |  |  |  |  |  |
| **RBC (unit, median (IQR))** | 4 (0-6) | 4 (2-8) | 4 (0-6) | 2 (0-6) | 2 (0-6) | <0.001 |
| **Plasma (ml, median (IQR))** | 400 (0-800) | 600 (0-800) | 400 (0-712) | 600 (0-800) | 400 (0-600) | 0.112 |
| **PLT (unit, median (IQR))** | 3 (1-4) | 4 (3-5) | 4 (3-4) | 1 (1-2) | 1 (1-2) | <0.001 |

*Penn Classification: Penn Class Aa (No ischemia), Penn Class Ab (Localized ischemia), Penn Class Ac (Generalized ischemia/circulatory collapse), Penn Class Ab&c (Combined ischemia). DHCA, deep hypothermic cardiac arrest; MHCA, moderate hypothermic cardiac arrest; SD, standard deviation; BMI, body mass index; COPD, chronic obstructive pulmonary disease; AD, aortic disease; Hx, history; HB, hemoglobin; WBC, white blood cell; PLT, platelet; TAR, total arch replacement; FET, frozen elephant trunk; ABO, aortic balloon occlusion; CABG, coronary artery bypass graft; CPB, cardiopulmonary bypass; HCA, hypothermic cardiac arrest; CMO, composite major outcomes; CRRT, continuous renal replacement therapy; ICU, intensive care unit; IQR, interquartile range; RBC, red blood cell; PLT, platelet.

**Supplemental Table 2. Preoperative, Operative and Outcome data by IQR after Weighted Propensity Score Matching**

|  | **Overall** | | **HCA_Q1 [13,19.6]** | **HCA_Q2 (19.6,23.1]** | **HCA_Q3 (23.1,25.5]** | **HCA_Q4 (25.5,29]** | **p value** |
| --- | --- | --- | --- | --- | --- | --- | --- |
| **n** | **612.7** | | **157.2** | **150.7** | **155.1** | **149.5** |  |
| **Preoperative characteristics** |  | |  |  |  |  |  |
| **Age (year, Mean±SD)** | 50.3±10.0 | | 49.9±8.8 | 50.8±10.1 | 50.6±9.6 | 49.9±11.4 | 0.684 |
| **Age>=60 year** | 112.2 (18.3) | | 21.1 (13.4) | 30.2 (20.0) | 28.1 (18.1) | 32.8 (22.0) | 0.137 |
| **Male (%)** | 455.2 (74.3) | | 119.4 (76.0) | 110.9 (73.6) | 115.7 (74.6) | 109.2 (73.0) | 0.909 |
| **BMI (kg/m^2, Mean±SD)** | 26.1±4.2 | | 26.1±4.2 | 26.2±4.2 | 26.2±4.2 | 26.1±4.3 | 0.979 |
| **Hypertension (%)** | 514.2 (83.9) | | 131.3 (83.5) | 127.5 (84.6) | 129.3 (83.4) | 126.0 (84.2) | 0.981 |
| **Diabetes Mellitus (%)** | 7.9 (1.3) | | 2.0 (1.3) | 2.0 (1.3) | 2.0 (1.3) | 1.9 (1.3) | 0.999 |
| **Corory Artery Disease (%)** | 16.7 (2.7) | | 5.1 (3.3) | 4.8 (3.2) | 3.8 (2.4) | 3.0 (2.0) | 0.825 |
| **COPD (%)** | 0.0 (0.0) | | 0.0 (0.0) | 0.0 (0.0) | 0.0 (0.0) | 0.0 (0.0) | 0.608 |
| **Marfan Syndrome (%)** | 27.1 (4.4) | | 6.9 (4.4) | 6.2 (4.1) | 7.2 (4.6) | 6.8 (4.5) | 0.986 |
| **Smoking (%)** | 264.9 (43.2) | | 66.2 (42.1) | 63.1 (41.8) | 69.6 (44.8) | 66.1 (44.2) | 0.903 |
| **Family Hx of AD (%)** | 5.6 (0.9) | | 2.2 (1.4) | 1.2 (0.8) | 1.6 (1.0) | 0.6 (0.4) | 0.576 |
| **Hx of Cardiac Surgery (%)** | 19.6 (3.2) | | 4.8 (3.0) | 5.5 (3.6) | 5.1 (3.3) | 4.3 (2.8) | 0.945 |
| **Hx of Aortic Surgery (%)** | 24.0 (3.9) | | 5.4 (3.4) | 7.0 (4.6) | 5.8 (3.7) | 5.8 (3.9) | 0.917 |
| **Onset within 14 days (%)** | 514.2 (83.9) | | 130.9 (83.2) | 125.9 (83.5) | 133.5 (86.0) | 124.0 (82.9) | 0.817 |
| **HB (g/L, Mean±SD)** | 135.2±16.8 | | 134.8±17.2 | 135.2±17.2 | 136.1±15.6 | 134.7±17.4 | 0.761 |
| **WBC (10^9/L, Mean±SD)** | 11.4±4.4 | | 11.3±3.7 | 11.3±5.4 | 11.6±3.9 | 11.4±4.5 | 0.804 |
| **PLT (10^9/L, Mean±SD)** | 192.3±74.9 | | 193.8±73.4 | 193.2±77.3 | 189.4±77.7 | 192.9±71.1 | 0.921 |
| **Penn Classification* (%)** |  | |  |  |  |  | 0.989 |
| **Penn Class Aa** | 477.1 (77.9) | | 119.8 (76.2) | 120.6 (80.0) | 120.1 (77.4) | 116.6 (78.0) |  |
| **Penn Class Ab** | 120.2 (19.6) | | 32.5 (20.7) | 26.6 (17.6) | 31.9 (20.5) | 29.2 (19.6) |  |
| **Penn Class Ac** | 13.4 (2.2) | | 4.3 (2.7) | 2.9 (2.0) | 2.9 (1.8) | 3.3 (2.2) |  |
| **Penn Class Ab&c** | 1.9 (0.3) | | 0.6 (0.4) | 0.6 (0.4) | 0.3 (0.2) | 0.3 (0.2) |  |
| **Operative data** |  | |  |  |  |  |  |
| **Root Operation (%)** |  | |  |  |  |  | 0.34 |
| **Bentall** | 128.3 (20.9) | | 34.0 (21.6) | 37.4 (24.8) | 32.8 (21.2) | 24.1 (16.1) |  |
| **Root-sparing** | 466.9 (76.2) | | 120.6 (76.7) | 111.1 (73.7) | 117.4 (75.7) | 117.9 (78.8) |  |
| **David** | 6.6 (1.1) | | 1.0 (0.6) | 0.7 (0.5) | 1.9 (1.2) | 3.0 (2.0) |  |
| **Wheat's** | 10.8 (1.8) | | 1.7 (1.1) | 1.6 (1.0) | 3.0 (1.9) | 4.6 (3.0) |  |
| **Arch Operation (%)** |  | |  |  |  |  | <0.001 |
| **TAR** | 12.5 (2.0) | | 4.5 (2.9) | 2.6 (1.7) | 4.5 (2.9) | 0.9 (0.6) |  |
| **TAR/FET** | 551.6 (90.0) | | 152.1 (96.8) | 144.9 (96.1) | 134.2 (86.5) | 120.4 (80.5) |  |
| **TAR/ABO** | 48.5 (7.9) | | 0.6 (0.4) | 3.2 (2.1) | 16.4 (10.6) | 28.3 (18.9) |  |
| **CABG (%)** | 67.4 (11.0) | | 22.3 (14.2) | 12.5 (8.3) | 12.8 (8.2) | 19.8 (13.3) | 0.104 |
| **Ascending-Iliac Bypass (%)** | 32.8 (5.4) | | 6.8 (4.3) | 7.1 (4.7) | 8.3 (5.4) | 10.6 (7.1) | 0.617 |
| **Operation Time (hour, Mean±SD)** | 6.2±1.7 | | 6.7±1.4 | 5.8±1.4 | 6.3±1.9 | 6.2±1.8 | <0.001 |
| **CPB Time (min, Mean±SD)** | 172.5±48.1 | | 189.9±48.7 | 166.9±39.7 | 162.6±47.1 | 170.2±51.5 | <0.001 |
| **HCA Time (min, Mean±SD)** | 18.3±7.3 | | 23.1±7.7 | 19.9±6.2 | 15.5±5.6 | 14.5±5.9 | <0.001 |
| **HCA Time>22 min (%)** | 139.8 (22.8) | | 76.1 (48.4) | 42.6 (28.3) | 13.4 (8.6) | 7.7 (5.2) | <0.001 |
| **Nadir Temperature (℃, Mean±SD)** | 22.7±3.5 | | 18.1±1.0 | 21.3±1.0 | 24.5±0.6 | 27.1±0.9 | <0.001 |
| **Clinical Outcomes** |  | |  |  |  |  |  |
| **CMO (%)** | 99.2 (16.2) | | 30.2 (19.2) | 27.7 (18.4) | 22.9 (14.8) | 18.4 (12.3) | 0.197 |
| **Operative Mortality (%)** | | 42.6 (6.9) | 16.0 (10.2) | 10.6 (7.1) | 9.1 (5.9) | 6.8 (4.5) | 0.125 |
| **Stroke (%)** | | 19.0 (3.1) | 4.2 (2.7) | 4.1 (2.7) | 5.2 (3.4) | 5.4 (3.6) | 0.933 |
| **Paraplegia (%)** | | 21.5 (3.5) | 3.3 (2.1) | 11.1 (7.4) | 4.7 (3.0) | 2.4 (1.6) | 0.004 |
| **CRRT (%)** | | 50.1 (8.2) | 18.2 (11.6) | 13.5 (9.0) | 8.7 (5.6) | 9.7 (6.5) | 0.104 |
| **Reexploration for bleeding (%)** | 24.1 (3.9) | | 6.9 (4.4) | 8.3 (5.5) | 6.2 (4.0) | 2.7 (1.8) | 0.236 |
| **Tracheotomy (%)** | 22.0 (3.6) | | 7.1 (4.5) | 4.5 (3.0) | 4.8 (3.1) | 5.6 (3.7) | 0.832 |
| **Hospital Stay (day, Mean±SD)** | 14.8±10.2 | | 17.3±15.6 | 14.5±8.4 | 13.6±6.5 | 13.6±6.9 | 0.002 |
| **>17 days (%)** | 149.3 (24.4) | | 51.4 (32.7) | 33.7 (22.3) | 32.4 (20.9) | 31.9 (21.3) | 0.014 |
| **ICU Stay (day, Mean±SD)** | 5.2±3.7 | | 5.7±2.9 | 5.6±3.1 | 4.4±3.4 | 5.1±4.9 | <0.001 |
| **>6 days (%)** | 154.7 (25.3) | | 52.1 (33.1) | 40.9 (27.1) | 29.4 (19.0) | 32.3 (21.6) | 0.005 |
| **Blood Loss (ml, Mean±SD)** | 885.7±475.9 | | 1014.3±612.6 | 862.6±401.1 | 848.2±352.0 | 812.4±469.6 | 0.001 |
| **In-Hospital Blood Product Use** |  | |  |  |  |  |  |
| **RBC (unit, Mean±SD)** | 5.3±7.2 | | 6.5±7.7 | 5.5±6.9 | 4.1±5.4 | 5.0±8.4 | 0.001 |
| **Plasma (ml, Mean±SD)** | 545.9±658.5 | | 610.5±664.7 | 484.0±590.6 | 573.3±599.5 | 512.1±763.4 | 0.182 |
| **PLT (unit, Mean±SD)** | 2.6±1.9 | | 4.1±1.6 | 3.4±1.5 | 1.7±1.4 | 1.4±1.4 | <0.001 |

*Penn Classification: Penn Class Aa (No ischemia), Penn Class Ab (Localized ischemia), Penn Class Ac (Generalized ischemia/circulatory collapse), Penn Class Ab&c (Combined ischemia). DHCA, deep hypothermic cardiac arrest; MHCA, moderate hypothermic cardiac arrest; SD, standard deviation; BMI, body mass index; COPD, chronic obstructive pulmonary disease; AD, aortic disease; Hx, history; HB, hemoglobin; WBC, white blood cell; PLT, platelet; TAR, total arch replacement; FET, frozen elephant trunk; ABO, aortic balloon occlusion; CABG, coronary artery bypass graft; CPB, cardiopulmonary bypass; HCA, hypothermic cardiac arrest; CMO, composite major outcomes; CRRT, continuous renal replacement therapy; ICU, intensive care unit; IQR, interquartile range; RBC, red blood cell; PLT, platelet.


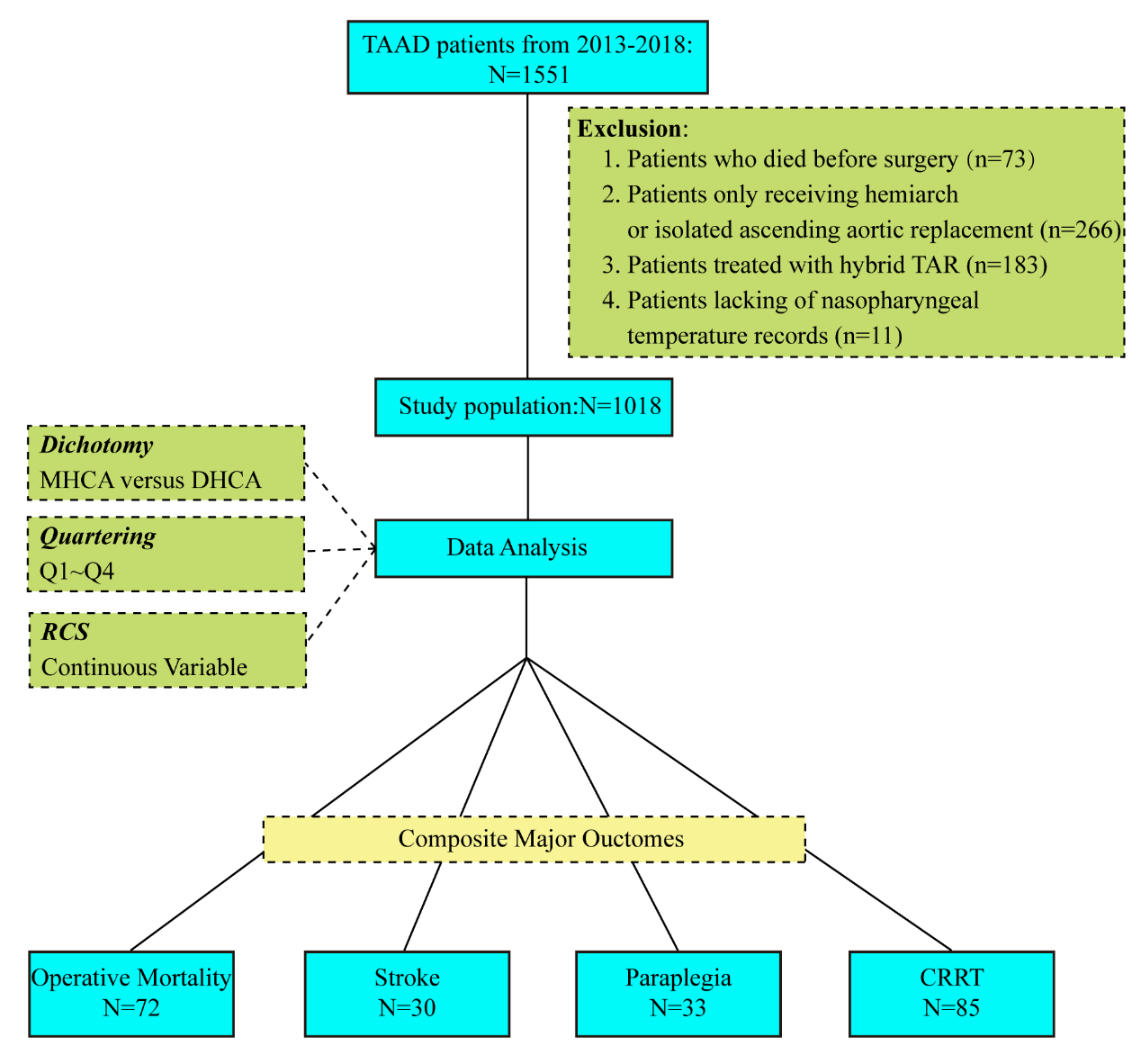


**Supplemental Figure 1**. Flow chart of the study. TAAD, type A aortic dissection; DHCA, deep hypothermic cardiac arrest; MHCA, moderate hypothermic cardiac arrest; RCS, restricted cubic spline; CRRT, continuous renal replacement therapy.


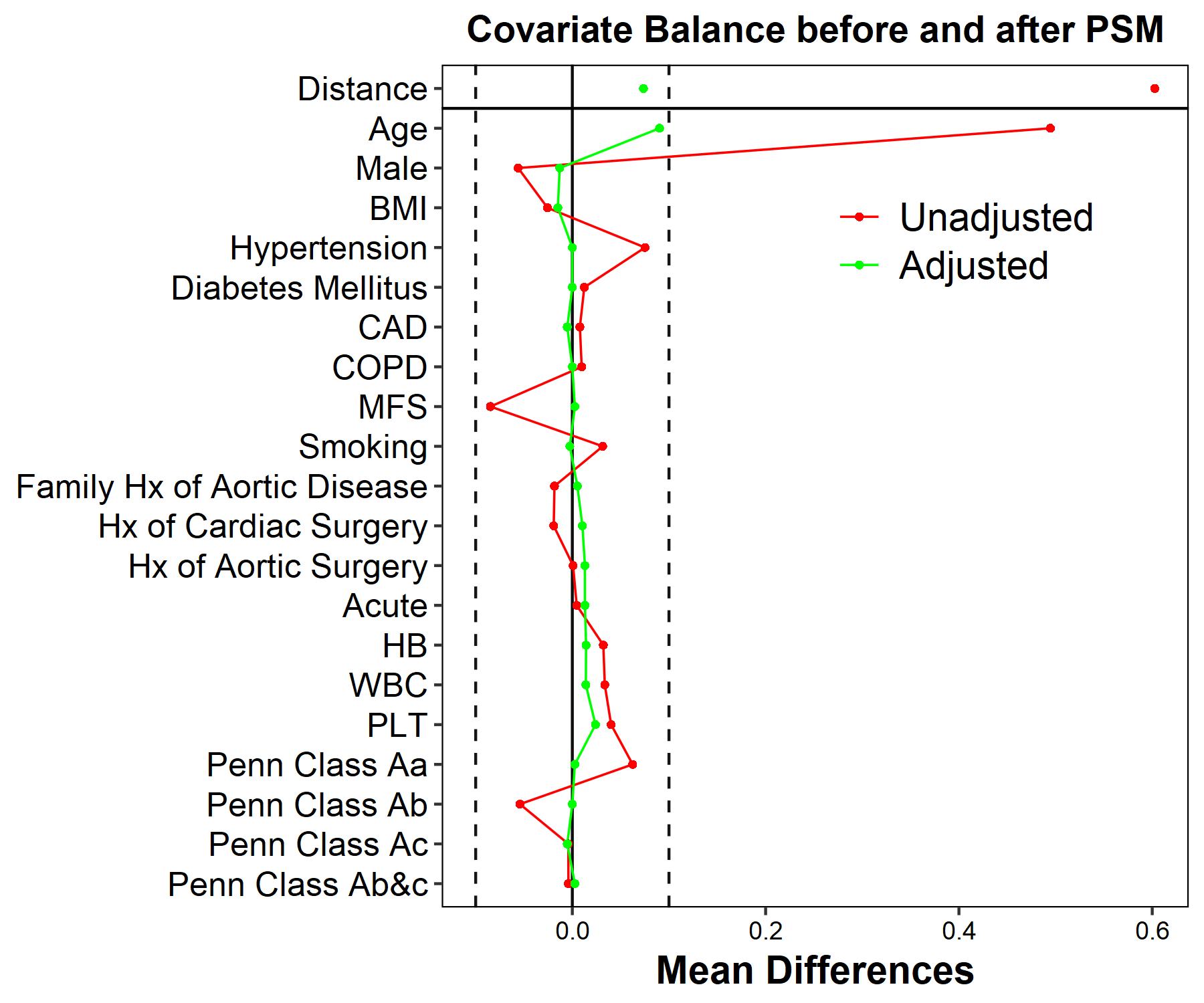


**Supplemental Figure 2**. Love plot for absolute standardized differences before (red line) and after (green line) propensity score matching comparing preoperative covariate values between DHCA and MHCA. PSM, propensity score matching; DHCA, deep hypothermic cardiac arrest; MHCA, moderate hypothermic cardiac arrest; BMI, body mass index; CAD, coronary artery disease; COPD, chronic obstructive pulmonary disease; MFS, Marfan syndrome; Hx, history; HB, hemoglobin; WBC, white blood cell; PLT, platelet.
